# *Photorhabdus* sp. ETL Antimicrobial Properties and Characterization of Its Secondary Metabolites by Gas Chromatography–Mass Spectrometry

**DOI:** 10.3390/life11080787

**Published:** 2021-08-04

**Authors:** Tshikala Eddie Lulamba, Ezekiel Green, Mahloro Hope Serepa-Dlamini

**Affiliations:** Department of Biotechnology and Food Technology, University of Johannesburg, Doornfontein Campus, P.O. Box 17011, Johannesburg 2028, South Africa; eddie_lulamba@yahoo.fr (T.E.L.); egreen@uj.ac.za (E.G.)

**Keywords:** *Photorhabdus heterorhabditis* strain ETL, secondary metabolites, antimicrobial properties, gas chromatography–mass spectrometry

## Abstract

Entomopathogenic nematodes (EPNs) are known to be highly pathogenic to insect pests, due to their associated symbiotic bacteria, which produce virulence factors, exo-enzymes and other harmful secondary metabolites to conquer, kill, and degrade their insect hosts. However, these properties are not fully characterized. This study reports on the antimicrobial activities of *Photorhabdus* sp. strain ETL, symbiotically associated to an insect pathogenic nematode, *Heterorhabditis zealandica*, against human pathogenic bacteria and toxigenic fungi, as well as the non-targeted profiling of its secondary metabolites (SMs) using gas chromatography coupled to high-resolution time-of-flight mass spectrometry. Fatty acids including 3-eicosene, (E)-; 5-eicosene, (E)-; eicosene; 9-octadecenamide; undecanoic acid with shown antimicrobial activities were detected. This provided more insight on the composition and bioactivities of SMs produced by the *Photorhabdus* sp.

## 1. Introduction

The entomopathogenic nematode bacteria (EPNB), *Xenorhabdus* and *Photorhabdus* (Enterobacteriales: Morganellaceae) [1], are known to produce inside their insects host an arsenal of virulence factors, antibiotic and exo-enzymatic compounds with pesticidal, pharmaceutical, and other beneficial properties [2,3,4,5,6]. These bioactive compounds have been grouped as bacteriocins, xenorhabdins, xenorxides, xenocoumacins, indole (including nematophin, genistein), stilbene and anthraquinone derivatives, and chitinases [2,3,7]. Therefore, EPNB have been considered among the major natural sources of novel bioactive molecules [3,8] that are important in the development of new drugs, agrochemicals, as well as food and biofuel industrial compounds [9,10,11].

Members of the *Photorhabdus* genus have an extensive secondary metabolism producing various secondary metabolites (SMs) that are thought to be virulent although their specific functions are not fully understood [4,12,13]. Secondary metabolism, although some SMs are produced during the exponential phase or bacterial growth, is generally activated during the post-exponential or stationary phase of the bacterial growth, which is during the mutual association with their nematode vectors [4]. Therefore, secondary metabolism, apart from producing anti-hosts effectors, is also important to support both nematode growth and development [4,6]. This was observed through the functions of some SMs such as multipotent stilbene (ST) antibiotics, antraquinone (AQ) and the ability to fluoresce, and through its ability to provide important nutrients to the nematode as large intracellular inclusions composed of crystal proteins with essential amino acids [4]. The ST antibiotic is required to initiate the recovery program of the nematode free-living life stage Infective Juvenile (IJ) during the mutual association [4], and during the nematode normal cycle of reproduction, the ST might act as a food signal stimulating the recovery of IJs, therefore functioning as a link between food availability with both nematode reproduction and development [4]. However, EPNB occurs in two phenotypic variants *viz.* primary and secondary variants, which differ according to morphology and SMs production [4,6]. The phenotypic primary variant has a normal secondary metabolism, and is the original bacterium variant, isolated from the EPN. In contrast, the secondary variant which is the resulting switching off of some secondary metabolism activities including the production of ST antibiotic, AQ pigment and the ability to fluoresce, and inefficiently produce intracellular inclusions, can arise probably to adapt to a life without the nematode in the environment [4,6]. Secondary variants can also arise after repeated in vivo and in vitro sub-culturing [14] during the bacterial stationary phase and when nematodes emerge from the cadaver [14,15], and this is influenced by nutrient limitation and redox stress [4,16,17]. This phenomenon of phenotypic variation is reversible with the *Xenorhabdus* and has not been reported for *Photorhabdus* spp. [14]. This phenomenon is critical bacterial SMs production because it cannot be manipulated during in vitro growth and a poor understanding of the biology of these bacteria can result in lower quality (variety and number) of produced SMs [14], in addition to other influencing factors, such us suitable medium, monoxenic conditions and adequate oxygen [15]. Secondary metabolites include numerous inert and mobile volatile low molecular weight natural products of diverse structures [18,19]. They are mainly divided into terpene, phenol and nitrogen containing compounds [20]. Volatile compounds serve to prevent or protect the host against pathogens [21].

A range of biochemical and genetic approaches can be utilized to identify and characterize these SMs. This is because some genetic loci that are involved in the production of these molecules are cryptic, therefore are not expressed under normal laboratory conditions [4]. Although the first natural products from *Photorhabdus* and other EPNB have been known for almost 30 years, a huge variety of new compounds have been identified in the last 5–10 years, mainly due to the application of modern mass spectrometry. Metabolomics has been recently implemented to advance the knowledge of the biology of both primary and secondary metabolites, and to identify new metabolites, as well as both their chemotaxonomy and alterations caused by them [22]. Mass spectrometry (MS) coupled with gas/liquid chromatography (GC-MS/LC-MS), or Nuclear Magnetic Resonance (NMR) analytical techniques, are generally used in metabolomics [23]. Liquid chromatography-MS can be used for unknown high molecular weight compounds, such as lipids, and for the profiling of low molecular weight compounds, such as saponins and phenylpropanoids [24], whereas gas chromatography–mass spectrometry (GC-MS) is used for low molecular weight compounds [25]. Non-volatile metabolites, not responding to the GC-MS approach, can be separated and characterized through reverse-phase column of the liquid chromatography–mass spectrometry (LC-MS) approach [20].

This study reports on the SMs produced in vitro by the South Africa-native EPNB, *P. heterorhabditis* strain ETL, using GC-MS, and analysis of its antimicrobial properties against selected human microbial pathogens and toxigenic fungi.

## 2. Materials and Methods

*Photorhabdus heterorhabditis* strain ETL conserved in glycerol stocks at −80 °C was reconstituted in nutrient agar (NA) (Merck KGaA, Darmstadt, Germany), supplemented with 0.025% bromothymol blue (MerckMillipore, Billerica, MA, USA) and 0.004% triphenyltetrazolium chloride (PanReac AppliChem, Barcelone, Spain), and MacConkey agar (Merck KGaA, Darmstadt, Germany). 

### 2.1. Selection of the Optimal Nutrient Medium for Metabolite Production

Five loopfuls of the log phase of *P. heterorhabditis* strain ETL obtained from NBTA agar plates were inoculated into 100 mL separate flasks containing 20 mL of Luria–Bertani broth (LB), nutrient broth (NB) (Neogen^®^ Culture Mediam, Lansing, MI, USA), NB supplemented with canola oil, and NB supplemented with canola oil and glucose [26]. The media were adjusted to final pH of 7.0 [27]. The flasks were simultaneously incubated at 28 °C for 48 h in the dark on an orbital shaker at 160 rpm. At one-hour intervals, the cell cultures were measured at 600 nm with an S-20 Boeco Spectrophotometer for 24 h. The media with highest bacterial yield was deduced to be the one suitable for the bacterial growth. This experiment was carried out in triplicate.

### 2.2. Extraction of Secondary Metabolites

Luria–Bertani broth (8 L) was prepared in Erlenmeyer flasks and autoclaved at 121 °C for 15 min. Flasks were inoculated with the *P. heterorhabditis* strain ETL and incubated in a shaking incubator at 125 rpm, at 30 °C for 7 days [28]. Thereafter, sterilized XAD-7-HP resin (SIGMA, South Africa, BCBR6696V) (20 g/L of the culture) was added and further shaken for 2 h. The resin was filtered through cheesecloth and eluted with acetone three times. Acetone was removed using a Rotary evaporator. The remaining water was extracted with ethyl acetate three times, using the separating funnel, and concentrated using a rotary evaporator [29]. The crude extract was re-suspended in 1 mL of methanol (chromatographic grade) in a dark amber vial for analysis after filtration.

### 2.3. Determination of Anti-Microbial Activity

#### 2.3.1. Minimum Inhibitory Concentrations (MIC)

The minimum inhibitory concentrations (MIC) and agar well diffusion methods were used to determine the antibacterial and antifungal activity of the *Photorhabdus heterorhabditis* strain ETL secondary metabolites. The following human pathogenic bacterial strains *Bacillus cereus* (ATCC 10876), *Enterococcus faecium* (ATTC 13048), *Escherichia coli* (ATCC 10536), *Klebsiella pneumonia* (ATCC 10031), *K. oxytoca* (ATCC 13182), *Pseudomonas aeruginosa* (NCTC 10662), *Staphylococcus* *aureus* (ATCC 25923), *S. saprophyticus* (ATCC 15305), *S. epidermidis* (ATCC 14990), *Veillonella parvula* (ATCC 10790), and *Mycobacterium smegmatis* (ATCC 21293), and fungal strains *Aspergillus flavus*, *A. niger*, and *A. parasiticus* obtained from the Food, Environment and Health Research Group (FEHRG) laboratories, Faculty of Health Sciences, University of Johannesburg, South Africa, were used. This was carried out following the methods outlined by Andrews [30], Rodriguez-Tudela [31], and Balouiri et al. [11]. Accordingly, the bacterial strains were inoculated in Mueller–Hinton broth and allowed to grow overnight in an incubator at 37 °C for 24 to 36 h, and compared to a 0.5 McFarland’s standard. Streptomycin standard solution (~1 mg/mL in 1 mM EDTA (Ethylenediamine tetraacetic acid) analytical grade) at 0.032 mg/mL (in sterile distilled water), was used as the positive control, while 0.1% DMSO (Dimethyl sulfoxide) was used as a negative control.

The crude secondary metabolite extract was weighed (0.173 g) into an empty sterile McCartney bottle and dissolved in 0.1% (*w*/*v*) DMSO to make a stock solution of 32 mg/mL. This solution was two-fold serially diluted using the Mueller–Hinton broth down to 0.03125 mg/mL. The 96 well microtiter plates were used. The outer wells were filled with sterile distilled water, while 100 µL of standardized bacterial cultures, grown overnight, were horizontally added into each inner well, with five repeats vertically. In vertical order, a decreasing concentration (from 16 to 0.03125 mg/mL) of 100 µL of the extract was added into each well. The plates were covered and incubated overnight at 37 °C. Thereafter, 10 µL of 0.02% (*w*/*v*) of Resazurin sodium salt solution was added, and further incubated for 2 h. Changes of colour from blue to pink to clear, upon reduction in the amount of oxygen within the medium, was observed as an indication of the microbial metabolism [32]. After a visual inspection for colour changes, the known metabolite concentration, in a well with slight colour change, in each horizontal row, was used as the MIC. The Agar disk-diffusion and Agar well diffusion methods were followed for fungal species.

#### 2.3.2. Agar Disk-Diffusion Method

The basic method, agar disk-diffusion, followed by the agar well diffusion method as described by Balouiri et al. [11] were used for the MIC testing, with minor modification. The toxigenic fungal strains *A. flavus*, *A. niger*, and *A. parasiticus* were used. Briefly, non-supplemented potato dextrose agar (PDA) plates were inoculated with the fungal strains adjusted to (0.4–5) × 10^6^ CFU/mL in potato dextrose broth (PDB). Five discs of filter paper (Whatman No. 1, Sigma Aldrich (Pty) Ltd., Jet Park 1459, South Africa) of about 6 mm in diameter, flooded with different concentrations of the crude secondary metabolite extract (two-fold serially diluted from 4 to 0.5 mg/mL), were placed on the agar surface then incubated at 36 °C for 5 days. Clotrimazole was used as the positive control. Thereafter, plates in triplicates were visually examined for the inoculum growth inhibition by the extract diffused within the agar. Diameters of zones of inhibition were compare with the CLSI (Clinical and Laboratory Standards Institute) standards. 

#### 2.3.3. Agar Well Diffusion Method

The agar well diffusion method by Balouiri et al. [11] was used for antifungal testing to better determine the bacterial zone of inhibition. Its procedure is similar to the disk-diffusion method, but for the inoculation, a volume of the microbial inoculum was spread over the entire agar surface. Then, 5 holes of a diameter ranging from 6 to 8 mm were punched aseptically using a sterile cork borer, and 20 to 100 µL of the crude secondary metabolite extract at 1 mg/mL was added into each well. A hole for Clotrimazole, used as positive control, was also punched. Then, plates were incubated at 36 °C for 5 days in triplicate. Secondary metabolites diffused in the medium were observed for growth inhibition of the tested fungal strains.

#### 2.3.4. Determination of Anti-Microbial Activity Methods’ Validation

The determination of anti-microbial activity methods was validated by a one-way analysis of variance (ANOVA) with Tukey and Duncan post hoc tests using IBM SPSS Statistics 26 (SPSS/IBM, Chicago, IL, USA); mean values of the repeated experiments (3×) were compared. Significant differences at the *p* ≤ 0.05 level of probability were then reported. 

### 2.4. Profiling of Volatile Compounds

The Pegasus gas chromatography (GC) coupled to a high-resolution time-of-flight mass spectrometry (MS) (GC-HRTOF-MS) system (LECO Corporation, St Joseph, MI, USA) equipped with an Agilent 7890A gas chromatograph (Agilent Technologies, Inc., Wilmington, DE, USA), a Gerstel MPS multipurpose autosampler (Gerstel Inc., Mülheim an der Ruhr, Germany) and a Rxi^®^-5 ms column (30 m × 0.25 mm ID × 0.25 μm) (Restek, Bellefonte, PA, USA) was used, operating in high-resolution. The instrument was successfully mass calibrated, prior to use, to assure accurate mass data collection. Mass calibration and pre-analysis calibration were performed using the compound perfluorotributylamine (PFTBA), and 11 masses including: CF_3_ (*m*/*z* 68.9952), C_2_F_4_ (*m*/*z* 99.9936), C_2_F_4_N (113.9967), C_2_F_5_ (*m*/*z* 130.9920), C_3_F_6_ (*m*/*z* 149.9904), C_4_F_9_ (*m*/*z* 218.9856), C_5_F_10_N (*m*/*z* 263.9871), C_8_F_16_N (*m*/*z* 413.9775), C_9_F_18_N (*m*/*z* 463.9743) and C_9_F_20_N (*m*/*z* 501.9711), respectively. The intensity and resolution values, 41392, 40200, respectively, as well as a mass accuracy RMS (root mean square) value <1 ppm, were observed.

One microliter of each sample was injected into the instrument in spitless mode and was carried by helium gas constantly pumped at 1 mL/min with inlet and transfer line temperatures of 250 and 225 °C, respectively. The oven was heated at successive ‘rests’ at increasing temperatures, designed to bake out the column. This consisted of an initial temperature of 70 °C, and 0.5 min rest; ramp up to 150 °C at 10 °C/min, and 2 min rest; and ramp up to 330 °C at 10 °C/min, and 3 min rest. The recommended rate and system extraction frequency was used for the MS data acquisition. This consisted of the rates of 13 spectra/s, 30 to 1000 *m*/*z*, and electron ionization and ion source temperature at 70 eV and 250 °C, respectively, and extraction frequency of 1.25 kHz. The ChromaTOF^®^ software was used for data analysis. The biological sample extraction was conducted in triplicate and each replicate analyzed twice (six analytical injections).

#### Data Processing and Statistical Analysis

The collected GC-HRTOF-MS dataset was converted to mzML format using the LECO ChromaTOF-HRT software and then processed (peak picking and alignment) on the XCMS open-source tool (https://xcmsonline.scripps.edu/, accessed on 19 October 2019). The resulting peak list variables, retention times (min), mass-to-charge ratios (*m*/*z*) and integrated peak areas were corrected. Statistically significant metabolites were identified based on their mass spectra and retention time using the National Institute of Standards and Technology (NIST), Mainlib and Feihn metabolomics libraries, and both online open chemistry databases and platforms, such as PubChem at the National Institutes of Health (NIH), Chemical Entities of Biological Interest (ChEBI) of the European Molecular Biology Laboratory-European Bioinformatics Institute (EMBL-EBI), and existing literature.

## 3. Results and Discussion

### 3.1. Selection of the Optimal Nutrient Medium for Metabolite Production

**Figure 1:**
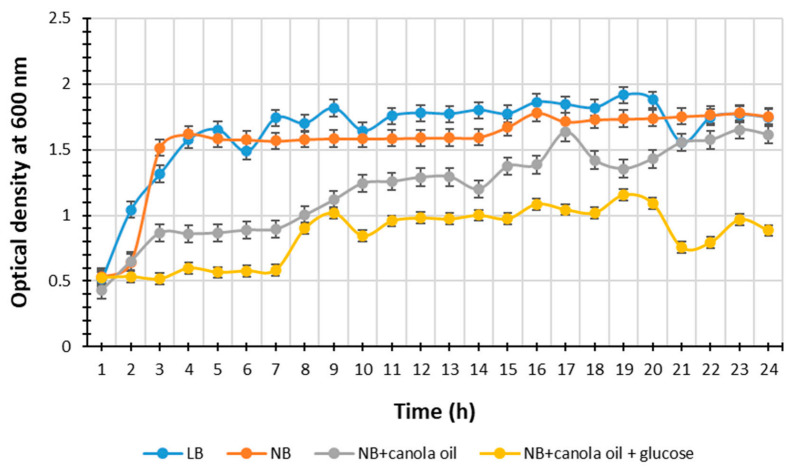
Absorbance over time at 600 nm of *P. heterorhabditis* strain ETL in Luria–Bertani broth, nutrient broth, nutrient broth supplemented with canola oil and nutrient broth supplemented with canola oil and glucose at approximately 25 °C on a shaker at 160 rpm for 24 h. Bars indicate the standard deviation of the repeated triplicates.

Distinctive color changes were observed (results not shown) in all media types, NB, NB with canola oil, NB with canola oil and glucose, and LB incubated with *P. heterorhabditis* strain ETL which indicated the secretion of secondary metabolites (SMs) by the bacterium as it proliferated and served as means to visually monitor the purity of the bacterial cultures, as some of these SMs have antibiotic activity [5] against opportunistic microorganisms that might contaminate the media. The results of *P. heterorhabditis* strain ETL growth are presented in Figure 1. The highest bacterial yields (Optical density, OD) value over the time (h) taken for the bacterial growth in LB broth, NB, NB supplemented with canola oil, and NB supplemented with canola oil and glucose was 1.9 at the 20th h, 1.8 at the 16th h, 1.6 at the 17th h, and 1.2 at the 19th h, respectively. 

The media type significantly affected the bacterial growth, and thus SMs secretion. The broth LB showed a highest bacterial yield, suggesting an increase in both bacterial growth and metabolism, due to nutrients availability, leading to an even increase in antibiotic production during the bacterial exponential growth phase, and to most SMs secretion during the bacterial stationery growth phase [4,33]. The NB medium also resulted in high microbial yield (Figure 1). These results are in accordance with the findings by Wang et al. [27], suggesting that these two media LB and NB are suitable for EPNB production because of the sodium chloride, component of the LB broth, which increases the metabolite production due to its osmolarity in the presence of good nitrogen sources [27]. The tryptone present in LB broth and both peptone and yeast extracts present in NB medium are good nitrogen sources [27].

### 3.2. Determination of Anti-Microbial Activity

Minimum Inhibitory Concentration (MIC)

The inhibition values of the crude extract of *P. heterorhabditis* strain ETL are presented in Table 1. The MIC values ranged from 4.0 to 0.0625 mg/mL. The crude extract MIC values at ≤1 mg/mL are considered active [34]. Thus, the observed inhibition values of up to 0.0625 mg/mL showed the potential of the SM to be used for new anti-microbial compounds development.

The extract from strain ETL had the most significant MIC values between 0.0625 and 1.000 mg/mL against *B. cereus*, *E. coli*, *K. oxytoca, K. pneumoniae*, *S. aureus*, *P. aeruginosa*, *S. epidermidis*, and *S. saprophyticus* species. The most significant MIC value of 0.0625 mg/mL was seen on pathogenic *E. coli*. Other notable inhibitory activities were seen on *S. saprophyticus* (0.125 mg/mL) and *M. smegmatis* and *S. aureus* (0.250 mg/mL). The crude extract from the strain ETL had higher fungal MIC properties (≤1 mg/mL of MIC) against *A. flavus* and *A. niger* than a crude extract from another reported EPNB (4 mg/mL ant 5 mg/mL for *A. flavus* and *A. niger*, respectively) [35], although a pur- antifungal compound (cyclo(l-Pro-d-Leu)) isolated from it showed greater MIC values of 0.008 mg/mL and 0.032 mg/mL against *A. flavus* and *A. niger*, respectively [35]. Members of the *Photorhabdus* produce numerous SMs with various antimicrobial activities [5,12] to suit the entomopathogenic lifestyle with their nematode vectors and to contribute to the symbiotic association with the nematode host by providing a monoxenic growth environment while killing off competitive microbes and insect larvae, and providing nutrients for both the nematodes and bacteria [4,36,37,38,39]. This could justify the notable inhibitory activity found in this study. Furthermore, the outer membrane in the Gram-negative organisms that is generally responsible for the more resistance to antibiotics than the Gram-positive bacteria, due to the exclusion of certain drugs and antibiotics from penetrating the cell [40], could also justify the fact that Gram-positive bacteria were more susceptible to the strain ETL crude extract’ SMs. These results suggest that strain ETL SMs can be further explored for the development of new pharmaceutical products against pathogens.

### 3.3. Overview and Exploration of the Acquired GC-MS Data

**Figure 2:**
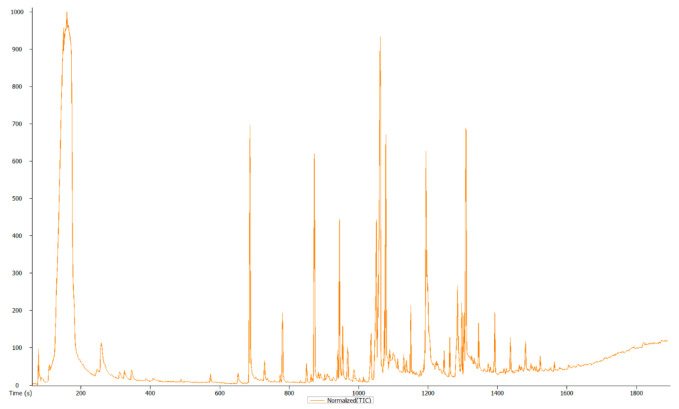
GC-MS chromatogram of *Photorhabdus heterorhabditis* strain ETL’s crude extract.

Considering the inherent chemo-diversity and multidimensionality of the extracted metabolome, the high-resolution GC-TOF-MS platform allowed the simultaneous detection of multiple analytes with high sensitivity, providing more detailed characterization of the metabolic inventory of the sample. Thus, visual inspection of the generated mass chromatogram pointed to 160 metabolic profiles of the analyzed sample, as shown in Figure 2. Table 2 presents a summary of the obtained precursors, fragments and retention time for the identified compounds, after the chromatogram peaks of the identified compounds being confirmed using online databases and the existing literature. 

About 22 volatile compounds, in various chemical groups, were identified from *P. heterorhabditis* strain ETL, as shown in Table 2. This number is below the number of natural products produced by members of the *Photorhabdus* genus [4]. This could be due to SMs’ cryptic genes not expressed under normal laboratory conditions [4], the unique extraction method [73], the media used for in vitro culturing, and both the unique approach and instrument used in this study. More SMs are still yet to be identified and characterized by using alternative methods. The strain ETL volatile compounds were found to be similar (or partly similar) to those produced by other pathogenic bacteria and fungi, including *Breibacillus brevis* (Bacillales: Paenibacillaceae), *Paracoccus pantotrophus* (Rhodobacterales: Rhodobacteraceae), *Streptomyces* sp. (Actinomycetales: Streptomycetaceae), *Aspergillus nidulans* (Eurotiales: Trichocomaceae) and *Lentinula edodes* (Agaricales: Omphalotaceae); and (medicinal) plants, including *Ammodaucus leucotricus* (Umbelliferae: Apiaceae), *Bidens pilosa* var. radiate (Malvales: Malvaceae), *Monochaetia kansensis* (Xylariales: Amphisphaeriaceae), *Rhaponticum acaule* (Asterales: Asterales), *Tamarix aphylla* L. (Caryophyllales: Tamaricaceae) and *Woodfordia Fruticosa* (L.) kurz (Myrtales:Lythraceae). However, no literature support was found regarding the tetradonium bromide and nonanamide compounds found in strain ETL. 

The functions and applications of the strain ETL-identified natural products are not fully understood; however, when reviewing the literature, valuable activities and putative roles in various industries were identified. Fatty acids including 3-eicosene, (E)-; 5-eicosene, (E)-; eicosene; 9-octadecenamide; undecanoic acid, methyl ester; benzenepropanoic acid, 3,5-bis(1,1-dimethylethyl)-4-hydroxy, methyl ester (FAME); and diethyl phthalate (FAEE) were detected among the strain ETL-produced natural products (Table 2). Most fatty acids, including FAMEs are recognized to have antifungal and antioxidant activities [74]. Some methyl esters (FAME), such as hexadecanoic acid, methyl ester, can be used as as biodiesel fuel [75] and FAME undecanoic acid, methyl ester as a component in lipases from *A. nidulans*, was found to have antimicrobial activities [66]. Lipases (and proteinases) have important functions in EPNB parasitism, as some are essential for insect colonization; they are involved in immunosuppression and tissue degradation [4,5,76,77]. Lipases in EPNB contribute efficiently in the degradation (and neutralization) of insect larvae adipose tissue which is a dynamic tissue implicated in many metabolic functions and is the main producer of key insect host proteins, such as both Pattern Recognition Proteins/Receptors (PRPs) and antimicrobial peptides (AMPs) [78,79,80]. The PRPs and AMPs are involved in either the humoral or the cellular immune response [5]. Thus, these lipases are an important source of energy and nutrients for both the bacterium and its nematode vector [5] and have been shown to contribute to host specificity through bioactivities against selected hosts [76].

The alcoholic compounds, 2-Pentanone, 4-hydroxy-4-methyl- (Table 2), have been found among the major components of essential oils of *A. leucotricus* (Umbelliferae: Apiaceae) to have a strong antibacterial activity against *S. aureus*, *E. coli* and *K. pneumonia* [41]. The bromomethane component of the tris (trifluoromethyl) bromomethane (Table 2) was produced both industrially and biologically and was used extensively as a pesticide until being phased out by most countries in the early 2000s [58,59]. The ketone compound, ergotaman-3′,6′,18-trione, 9,10-dihydro-12′-hydroxy-2′-methyl-5′(phenylmethyl), (5′à,10à)- was found to have a biocontrol activity against *Lasiodiplodia theobromae* (Botryosphaeriales: Botryosphaeriaceae)*,* a plant pathogen causing black spot disease on wax apple and is reportedly a potential biocontrol agent against pathogens on fruits [71]. Diethyl phthalate derivatives showed antimicrobial activity against *A. niger*, *A. flavus*, *Trichoderma harizanum* (Hypocreales: Hypocreaceae), *T. viridae* and *Rhizoctonia solani* (Cantharellales: Ceratobasidiaceae) [81]. Pyrrolo[1,2-a]pyrazine-1,4-dione, hexahydro-3-(2-methylpropyl)-, having antioxidant properties and antimicrobial activities (Table 2), might reportedly have a potential future in agriculture [62]. The benzenepropanoic acid (carboxylic acid) component of the benzenepropanoic acid, 3,5-bis(1,1-dimethylethyl)-4-hydroxy-, methyl ester (Table 2), as with other members of the phenylpropanoids class, are used in food as antioxidants, sweeteners, emulsifiers, and flavorings for ice cream, bakery, and confectionary. In cosmetics, they are used as a flagrant agent giving a floral scent to bath gels, detergents (both liquid and powders), fabric softeners, perfumes and soaps [63]. The diethyl phthalate (Table 2) can be useful in the development of useful agricultural, pharmaceutical and industrial products. Finally, tridecane is used for the development of solvents and fuels [42,43], indicating the diverse usefulness of the SMs from *Photorhabdus*, not only in pharmaceutical but other industries as well. These volatile organic compounds showed the diverse useful applications of the SMs from *P. heterorhabditis* strain ETL and can possibly play a critical role in the food, cosmetic and drug discovery industries. Despite these findings, some of these produced natural products including their physiological and/or biological role(s) are still not fully clear. This is because the inference of roles for some natural products is not as easy as it is for antibiotically active natural products in the bacterial ecosystem, and antibiotics in sub-inhibitory concentrations induce changes in both the mRNA and the levels of proteins produced in other bacteria [82,83], thus serving as signal molecules in natural environments. Therefore, future studies to determine the genetic characteristics and mechanisms involved in the *P. heterorhabditis* strain ETL SMs production, as well as the genetic evolutionary differences and similarities with other EPNB are needed. 

## 4. Conclusions

The EPNB produce numerous SMs with various bioactivities, and their functions are not fully understood. Nevertheless, evidence shows that *P. heterorhabditis* strain ETL and other EPNB may provide substantial benefits to agriculture, pharmaceutical industries and the environment. In brief, this study determined the antimicrobial activity of the crude extracts of the EPNB, *P. heterorhabditis*, which showed notable inhibitory activities. The antibacterial results show the potential use of this bacterium for the isolation of pure bioactive compounds and possible drug discovery. Additionally, a useful fingerprint on major volatile SMs produced by *P. heterorhabditis* is provided. Bioactive compounds with pesticidal, pharmaceutical, medicinal, agricultural, food and cosmetic industrial potentials were identified. This thus provided more insight on the composition and bioactivities of SMs produced by the *P. heterorhabditis* strain ETL.

## Figures and Tables

**Table 1:** Minimum inhibitory concentration (MIC) and both Agar disk-diffusion and Agar well diffusion methods values of the antimicrobial activity tests carried out on the secondary metabolites from *Photorhabdus heterorhabditis* strain ETL.

Test Organism	Gram Reaction of Test Microorganisms	MIC (mg/mL)	Positive Control (Streptomycin) (mg/mL)
**Bacteria**			
*Pseudomonas aeruginosa*	Negative	0.83 ± 0.28 ^cd^	0.025
*Klebsiella oxytoca*	Negative	0.42 ± 0.14 ^ab^	1
*Escherichia coli*	Negative	0.062 ± 0 ^a^	1
*Staphylococcus aureus*	Positive	0.25 ± 0 ^ab^	<0.031
*Staphylococcus epidermidis*	Positive	1.00 ± 0 ^d^	1.000
*Klebsiella pneumoniae*	Negative	0.42 ± 0.14 ^ab^	2.000
*Veillonella parvula*	Negative	4.00 ± 0 ^e^	0.062
*Enterococcus faecium*	Positive	4.00 ± 0 ^e^	0.025
*Bacillus cereus*	Positive	0.500 ± 0 ^bc^	0.5
*Staphylococcus saprophyticus*	Positive	0125 ± 0 ^a^	0.062
*Mycobacterium smegmatis*	Positive	0.25 ± 0 ^ab^	0.5
**Fungi**	**Fungal MIC** **(mg/mL)**	**ZI** **(diameter in mm)**	**Clotrimazole**
*Aspergillus flavus*	1.00 ± 0 ^d^	14 ± 1.2	1
*Aspergillus niger*	0.83 ± 0.28 ^cd^	12 ± 1	1
*Aspergillus parasiticus*	1.00 ± 0 ^d^	12 ± 1.3	1

^a,b,c,d,e^ Mean values in the same column not sharing the same superscript are significantly different (*p* < 0.05). Values within columns are means (left) and standard deviation (right), and ZI: zone of inhibition.

**Table 2:** Summary of the precursors, fragments and retention time for significant compounds identified in *Photorhabdus heterorhabditis* strain ETL’s crude extract.

	R_t_ (s)	*m*/*z*	Actual Masses	MF	Name	MC/Compound Nature	Activity/Function (References)
1.	159.547	103.0652	43.018008	C_6_H_12_O_2_	2-Pentanone, 4-hydroxy-4-methyl-	Alcohol	Strong antibacterial activity [41].
2.	324.79	131.124	43.054469	C_13_H_28_	Tridecane	Long-chain alkane	Volatile oil component of various fuels and solvents; a distillation chaser in research laboratories [42,43].
3.	702.79	206.1668	191.143147	C_14_H_22_O	Phenol, 2,5-bis(1,1-dimethylethyl)-	Phenol/Aromatic hydro carbon	Antibacterial activity [44].
4.	780.266	141.1627	57.070034	C_16_H_34_	Hexadecane	Alkane long chain hydrocarbon	Antimicrobial and antioxidant activity [45].
5.	782.647	202.1097	149.023421	C_12_H_14_O_4_	Diethyl Phthalate	Diester of phthalic acid (FAEE), ethyl ester, Phthalate ester	Antimicrobial activity, a teratogenic agent, neurotoxin, plasticiser, and an endocrine disruptor [46,47,48,49,50,51,52,53,54,55].
6.	849.532	147.0928	43.054409	C_19_H_39_Cl	Nonadecane, 1-chloro-	Alkane (long-chain)	Antioxidant [56].
7.	871.555	241.2753	58.065283	C_17_H_38_BrN	Tetradonium Bromide	Nitrogen compound (germicidal detergent)	-
8.	883.831	140.1559	57.070068	C_22_H_42_O_4_	Oxalic acid, isobutyl hexadecyl ester	Ester, Organic acid	Oxalic acid has antimicrobial activity [57].
9.	910.782	131.1138	69.057689	C_4_BrF_9_	Tris(trifluoromethyl) bromomethane	Bromomethane (or methyl bromide)/organobromine compound.	Pesticide [58,59].
10	939.259	131.0866	43.054488	C_18_H_36_	3-Octadecene, (E)-	Alkene	Antimicrobial activity [60].
11	966.787	163.0868	70.065210	C_11_H_18_N_2_O_2_	Pyrrolo[1,2-a]pyrazine-1,4-dione, hexahydro-3-(2-methylpropyl)-	Antibiotic compound	Antioxidant properties, antimicrobial activity [40,61,62].
12.	1046.92	292.2021	57.070074	C_18_H_28_O_3_	Benzenepropanoic acid, 3,5-bis(1,1-dimethylethyl)-4-hydroxy-, methyl ester	Aromatic acid ester (FAME)/Benzenepropanoic acid (carboxylic acid)	Fixative, or preservative agents used in foods, cosmetics, and medicines [63].
13.	1060.08	233.1522	149.023469	C_25_H_40_O_4_	Phthalic acid, 6-ethyl-3-octyl heptyl ester	Ester/Plasticizer Compound	Antimicrobial, antifouling [64].
14.	1066.93	124.1123	59.036837	C_9_H_19_NO	Nonanamide	Amide	-
15.	1073.98	139.1472	43.054469	C_20_H_40_	3-Eicosene, (E)-	Alkene/Long chain fatty acid	Antibacterial activity [44,65].
16.	1074.22	192.982	55.054478	C_20_H_40_	5-Eicosene, (E)-	Alkene/Long chain fatty acid	[44,65].
17.	1077.64	183.2102	57.070030	C_20_H_42_	Eicosane	Alkane/Long chain fatty acid	Antibacterial activity [44,65].
18.	1153.56	199.1687	74.036306	C_12_H_24_O_2_	Undecanoic acid, methyl ester	FAME/Medium-chain fatty acids	Antimicrobial, antioxidant [66,67].
19.	1284.1	262.2527	59.036757	C_18_H_35_NO	9-Octadecenamide	Amide/Fatty acid amide	Anti-inflammatory and antibacterial. activities [68,69].
20.	1296.83	219.0448	57.070020	C_27_H_56_	Heptacosane	Alkane	Insecticidal activity [70].
21.	1302.48	244.1204	70.065251	C_33_H_37_N_5_O_5_	Ergotaman-3—,6′,18-trione, 9,10-dihydro-12′-hydroxy-2′-methyl-5′-(phenylmethyl)-, (5′a,10a)-	Ketone	Antimicrobial (antifungal) [71].
22.	1437	225.2612	BPI(57.070047)	C_28_H_58_	Octacosane	Alkane	Insecticidal activity [72].

FAEE—fatty acid ethyl ester, FAME—Fatty acid methyl esters, *m*/*z*—mass-to-charge ratio, MC—metabolite class, MF—molecular formula, Rt—retention time (s).

## Data Availability

None.
